# Investigating the direct and indirect associations between birth intervals and child growth and development: A cross-sectional analysis of 13 Demographic and Health Surveys

**DOI:** 10.1016/j.ssmph.2022.101168

**Published:** 2022-07-09

**Authors:** Lilia Bliznashka, Joshua Jeong

**Affiliations:** a Global Academy of Agriculture and Food Systems, University of Edinburgh, United Kingdom; b Department of Global Health and Population, Harvard T.H. Chan School of Public Health, Boston, MA, USA

**Keywords:** Low- and middle-income countries, Preschoolers, Birth spacing

## Abstract

There is considerable literature on the associations of short birth intervals with adverse perinatal outcomes. However, less is known about the associations with child growth and development. In this study, we investigated the associations between birth intervals and child growth and development and examined child illness, child diet, and maternal stimulation as potential mechanisms. We pooled Demographic and Health Survey data on 8300 children aged 36–59 months from 13 countries (Benin, Burundi, Cambodia, Cameroon, Chad, Congo, Haiti, Honduras, Rwanda, Senegal, Timor-Leste, Togo, and Uganda). Longer birth interval was defined as a preceding birth interval ≥33 months. Child growth was assessed using height-for-age Z-score (HAZ). Child cognitive and socio-emotional development were measured using the Early Childhood Development Index. Child morbidity was defined as any illness in the past two weeks. Child diet was assessed using dietary diversity score and maternal stimulation by the number of stimulation activities. We used generalised linear models to estimate associations between longer birth intervals and child growth and development. Structural equation modelling was used to assess direct and indirect effects. In our sample, 44% of children had a preceding birth interval ≥33 months, 42% were stunted, 25% were cognitively off-track, and 33% socio-emotionally off-track. Longer birth intervals were associated with higher HAZ (mean difference 0.23 (95% CI 0.14, 0.32)) and socio-emotional development (relative risk (RR) 1.04 (95% CI 1.00, 1.09), but not cognitive development (RR 1.02 (95% CI 0.98, 1.06). We observed no significant indirect effects via child illness, child dietary diversity, or maternal stimulation. Although longer birth intervals were beneficial for child growth and socio-emotional development, we found no empirical support for the biological and behavioural mechanisms we explored. Additional research is needed to investigate alternative mechanisms to elucidate underlying processes and inform future interventions.

## Highlights

• Longer birth intervals were associated with improved child growth and development.
• Longer birth intervals were more beneficial for child outcomes in wealthier households.
• Child illness, diet, and stimulation were not empirically substantiated as mechanisms.
• Future research could explore additional biological and behavioural mechanisms.

## 1 Introduction

In low- and lower-middle-income countries (LLMICs), fertility rates remain high at an average of 4.6 births per woman in low-income countries and 2.7 births per woman in lower-middle income countries (World Bank, 2019). High fertility rates often lead to shorter birth intervals (Cleland et al., 2012), which are associated with adverse maternal and child perinatal health outcomes in the first year of life, including preterm birth, low birth weight, and infant mortality (Bauserman et al., 2020; Conde-Agudelo et al., 2006).

A growing number of studies in LLMICs have also shown that short birth intervals continue to compromise children's growth and development beyond infancy and throughout early childhood. Specifically, shorter birth intervals are associated with an increased risk of stunting and underweight in children under five years of age (Dewey & Cohen, 2007; Fink et al., 2014; Rutstein, 2005; Shifti et al., 2022; Yaya et al., 2020). However, the relationship between birth intervals and child cognitive and socio-emotional development has received much less attention. Nascent evidence from high-income countries suggests that shorter birth intervals are associated with poor cognitive and behavioural development in pre-schoolers (Crowne et al., 2012; El-Kamary, 2004) and with an increased risk of autism spectrum disorder in one study among children under five years of age (Conde-Agudelo et al., 2016).

The mechanisms linking short birth intervals and child growth and development generally fall within two categories: (1) maternal physiological and biological mechanisms, and (2) behavioural mechanisms (Miller & Karra, 2020). The former include mechanisms such as maternal nutritional depletion, increased cervical insufficiency, and vertical infection transmission, and primarily explain the associations between short birth intervals and adverse maternal perinatal and child health and nutrition outcomes (Conde-Agudelo et al., 2012; Dewey & Cohen, 2007). In addition to vertical infection transmission, horizontal transmission of infections between siblings may also mediate the associations between short birth intervals and child health outcomes (Conde-Agudelo et al., 2012; Miller & Karra, 2020). Empirical studies have documented that shorter birth intervals are associated with increased morbidities among children under five years of age, including acute respiratory illness, diarrhoea, and fever (Fagbamigbe et al., 2021; Rahman et al., 2019). Although these studies lend support to the infection transmission mechanism, none of them explicitly tested it as a mediator.

In contrast, behavioural mechanisms between short birth intervals and child outcomes are hypothesised to operate through parental investment and sibling competition for parental resources, care, and attention (Conde-Agudelo et al., 2012; Miller & Karra, 2020). Prior studies have examined the sibling competition hypothesis through the survival status of the previous sibling (Conde-Agudelo et al., 2012; DaVanzo et al., 2004). However, to our knowledge, no studies have tested parental investment and sibling competition with regards to other resources, such as food and parental stimulation, as possible mechanisms. Parental behavioural mechanisms may potentially have a unique role in explaining the relationship between short birth intervals and child outcomes during the early childhood period, when children increasingly seek inputs from caregivers (Vygotsky, 1978).

Given the lack of evidence on the relationship between birth spacing and child development in LLMICs and the theoretically plausible role of biological and behavioural mechanisms in explaining this relationship, the objective of this study was twofold: (1) to investigate the relationship between birth spacing and child growth and development in children 36–59 months of age in LLMICs, and (2) to examine child illness, child diet, and maternal stimulation as potential mechanisms. Our study contributes to a limited literature on the intergenerational links between birth intervals and child growth and development and expands it by assessing these associations in 13 LLMICs. Our study further extends the global evidence on older preschoolers 36–59 months of age, an often overlooked age group due to studies and interventions focusing on the first 1000 days (from conception to two years of age) as a particularly sensitive period for child growth and development (Kerac, 2022). Our findings can help inform programmes to support the development, growth, and health of older preschoolers in LLMICs.

## 2 Methods

### 2.1 Conceptual model

**Fig. 1:**
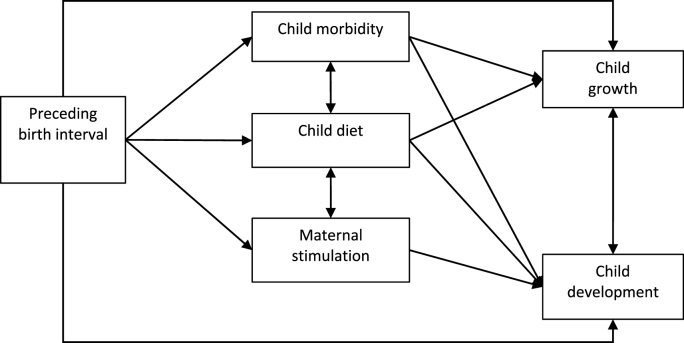
Conceptual model of the relations between birth intervals and child growth and development. Bidirectional arrows represent correlations.

Drawing from the All Children Surviving and Thriving Framework (Black et al., 2020), we applied a multi-dimensional caregiving perspective to our investigation of the relationship between birth spacing and child growth and development. Fig. 1 presents our conceptual model. Based on the literature presented above, we hypothesised positive direct effects between longer birth intervals and the two outcomes of child growth and development. With respect to the three mechanisms, we hypothesised positive direct effects between longer birth intervals and child diet and maternal stimulation, and a negative direct effect between longer birth intervals and child morbidity. In addition, we hypothesised that each of these mechanisms could mediate effects with child growth and development. Specifically, we hypothesised a negative indirect effect between longer birth intervals and child growth and development through child illness and positive indirect effects between longer birth intervals and child growth and development through child diet and maternal stimulation. Longer birth intervals could improve child diet by reducing the number of older siblings directly competing for food resources in the households (i.e., older siblings may be receiving (pre-)school meals) and thus improving food availability and accessibility for their younger siblings at home. Likewise, with respect to maternal stimulation, longer birth intervals imply fewer young children in the household, which in turn may increase caregivers’ attention and resources available for an individual child. For example, caregivers with fewer young children may be less preoccupied with childcare and spend less time on household chores and responsibilities and thus have more time to provide stimulation.

### 2.2 Data and study population

Similar to previously published articles (Bliznashka et al., 2021), we pooled data from the latest Demographic and Health Surveys (DHS) for 13 LLMICs countries: Benin, Burundi, Cambodia, Cameroon, Chad, Congo, Haiti, Honduras, Rwanda, Senegal, Timor-Leste, Togo, and Uganda (Supplemental Table 1). These 13 countries represented all countries which collected data on child development, diet, and stimulation and were publicly available as of September 2021. Child development and stimulation are collected in the optional early childhood development module. This module is applied to the youngest child aged 36–59 months only. It includes questions about maternal stimulation, attendance of early childhood care and education programmes, and children's attainment of developmental milestones. Since the early childhood development module is optional in DHS, it is available for a limited number of countries. In households with children 36–59 months of age, questions on dietary intake are administered for one randomly selected child in this age group.

### 2.3 Measures

The preceding birth interval was defined as the length in months between the birth date of the child under study and the birth date of the preceding child (Croft et al., 2018). We created a binary variable for longer birth interval, which indicated whether or not the preceding birth interval was ≥33 months (i.e., at least 33 months between live births), consistent with the World Health Organization (WHO) recommendation for at least 24 months between a live birth and the next pregnancy (World Health Organization Department of Reproductive Health and Research, 2007).

Child growth was assessed using height-for-age Z-score (HAZ), calculated using the 2006 WHO child growth standards (World Health Organization, 2006). We focused on linear growth (instead of ponderal growth) as a marker of chronic deprivation, which at least partially shares determinants with child development (Prado et al., 2019). Most prior empirical literature on birth intervals has found stronger associations with linear growth (Dewey & Cohen, 2007; Fink et al., 2014; Rutstein, 2005; Shifti et al., 2022) and therefore there was stronger evidence for examining mechanisms with respect to linear as opposed to ponderal growth. Because of the many limitations of stunting as an indicator of child health (Leroy & Frongillo, 2019; Perumal et al., 2018) and the fact that nutrition interventions for children 36–59 months of age would likely result only in minor changes in child height (Perumal et al., 2018), we focused on the continuous HAZ indictor in our analyses. By DHS design, the Early Childhood Development Index (ECDI) is collected from the youngest child aged 36–59 months in each households. Mothers reported on whether their child attained each one of ten development milestones. Based on prior work (McCoy et al., 2016), we focused on cognitive (two items for whether the child follows simple directions on how to do something correctly and whether the child is able to do something independently when given something to do) and socioemotional development (three items for whether the child gets along well with other children, whether the child kicks, bites or hits other children, and whether the child gets easily distracted). We created binary indicators for whether children were developmentally on-track in each domain, defined as children achieving at least one milestone in the cognitive domain and at least two milestones in the socio-emotional domain (Loizillon et al., 2017).

Child morbidity was assessed based on maternal recall of whether the child had any illness (diarrhoea, cough, or fever) in the two weeks prior to the survey. Child diet was assessed using the WHO indicator for dietary diversity score (DDS, range 0–7). Mothers reported the food groups consumed by the child in the 24 h prior to the survey (World Health Organization and United Nations Children’ s Fund, 2021). DDS was originally validated for children <24 months of age (World Health Organization and United Nations Children’ s Fund, 2021). However, one recent study showed that DDS is also a valid proxy for micronutrient adequacy in children 24–59 months of age (Diop et al., 2021). To assess maternal stimulation we summed the number of activities the mother engaged in with the child in the three days prior to the survey (range 0–6). Mothers reported on their engagement in the following activities: reading books or looking at pictures, telling stories, naming/counting/drawing, singing, taking the child outside, and playing with the child.

### 2.4 Statistical analysis

We restricted the analytic sample to children 36–59 months of age with available data on preceding birth interval, diet, and development. First, we used generalised linear models to examine the associations between longer birth intervals and child growth and development. We used a linear model for HAZ and calculated unadjusted and adjusted mean differences (MD). The adjusted linear model took the following form:

(1) $$HAZ_{i}=\beta_{0}+\beta_{1}BI_{i}+\beta_{3}\bar{X_{i}}+\varepsilon_{i}$$

where $HAZ_{i}$ is the observed HAZ for child *i*, $BI_{i}$ is the observed preceeding birth interval for child *i,* $\bar{X_{i}}$ is a vector of household, maternal, and child characteristics, and $\varepsilon_{i}$ is the error term. We used a log-Poisson model for the binary indicators for on-track child cognitive and socio-emotional development and estimated unadjusted and adjusted relative risks (RR). The adjusted log-Poisson model took the following form:

(2) $$\log\left(\;\mu_{i}\right)=\beta_{0}+\beta_{1}BI_{i}+\beta_{3}\bar{X_{i}}$$

$$Y_{i}\;∼\;Poisson\left(\mu_{i}\right)$$

where $Y_{i}$ is the observed binary indicator for on-track cognitive or socio-emotional development for child *i*, which has a Poisson distribution with a mean $\mu_{i}$, $BI_{i}$ is the observed preceeding birth interval for child *i,* and $\bar{X_{i}}$ is a vector of household, maternal, and child characteristics. Since we were interested in conducting multigroup analysis and we had a common outcome, we estimated relative risks instead of odds ratios because odds ratios are non-collapsible, meaning the population odds ratio is not a weighted average of the sub-group odds ratios (Hernan & Robins, 2020). Poisson models provide a correct estimate of relative risk under equal follow-up time (McNutt et al., 2003). However, for common outcomes (like in our case), Poisson models provide less precise confidence intervals than log-binomial models (McNutt et al., 2003). The vector of household, maternal, and child characteristics in adjusted models included: household wealth, location (urban vs. rural), size, access to improved sanitation, and gender of the household head; maternal age, education, and marital status; child age, sex, whether the child had a twin, number of siblings, child illness and child diet. The models for cognitive and socio-emotional development also controlled for maternal stimulation. The models were weighted for representativeness by applying DHS sampling weights. SEs were clustered at the primary sampling unit level.

Second, we used structural equation modelling (Kline, 2015) to test the conceptual model presented in Fig. 1 and examine the direct and indirect effects between birth intervals and child growth and development via child illness, child diet, and maternal stimulation. Given the cross-sectional nature of our data, we could not estimate causal effects. Therefore, the “direct effects”, “indirect effects”, and “total effects” we refer to are standard nomenclature from the structural equation modelling and path analysis literature (Kline, 2015). All direct paths controlled for household wealth, location, size, access to improved sanitation, and gender of the household head; maternal age, education, and marital status; child age, sex, whether the child has a twin, and number of siblings. Missing data on child illness and maternal stimulation were handled by using a full weight matrix. Missing data on control variables (N = 10) were imputed using mean imputation. The structural equation model also accounted for clustering and representativeness. Absolute model fit was assessed using Comparative Fit Index (CFI), Root Mean Square Error of Approximation (RMSEA), and Standardized Root Mean Squared Residual (SRMR) and determined acceptable if CFI ≥0.90, RMSEA ≤0.08, and SRMR ≤0.08 (Hu & Bentler, 1999). We used bootstrapping with 5000 draws to calculate bias-corrected (BC) bootstrapped 95% confidence intervals (CI) to test the significance of the total, direct and indirect effects (Preacher & Hayes, 2008). We refer to these as 95% CI for brevity.

Lastly, we conducted exploratory multigroup analyses to determine if the parameters of the structural equation model differed by maternal education (mother had some education vs. mother had no education) and household wealth (household was in the lowest two wealth quintiles vs. household was in the highest three wealth quintiles). To test for invariance between groups, we conducted a Wald test for difference between a model where all parameters were constrained to be equal across groups and a model where all parameters were unconstrained across groups. Differences between groups were considered significant at p < 0.05. We did not control for survey country or year in any of the analyses since we hypothesised that the associations of interest would not systematically differ by these two characteristics. The descriptive analyses and generalised linear models were estimated in Stata 17 (StataCorp, 2021). The structural equation model, model fit statistics, and multigroup analyses were estimated in MPlus Version 8.3 (Muthén & Muthén, 2017).

## 3 Results

Our analytic sample included 8300 children 36–59 months of age (Table 1). The preceding birth interval was ≥33 months for 44% of children. Child growth and development were generally poor with 42% of children stunted, 25% cognitively off-track, and 33% socio-emotionally off-track. Child dietary diversity and maternal stimulation were also suboptimal, and child illness was common.

**Table 1:** Household, maternal, and child characteristics of the 8300 children in the analytic sample.

	Mean (SD) or Proportion
*Household characteristics*	
Size, number of household members	8.3 ± 4.4
Lives in rural area	75.9
Is in lowest two wealth quintile	53.1
Has access to improved sanitation	22.9
*Mother characteristics*	
Age, years	31.0 ± 5.6
Highest level of education	
No education	50.8
Primary education	35.3
Secondary or higher education	14.0
Married or cohabitating	96.4
*Child characteristics*	
Male	50.9
Age, months	47.1 ± 6.8
Preceding birth interval was ≥33 months	43.8
Has at least one younger sibling	4.9
Cognitive development off-track	25.5
Socio-emotional development off-track	33.1
Height-for-age Z-score	−1.9 ± 1.4
Stunted (height-for-age Z-score <2 SD)	42.4
Dietary diversity score in the past 24 h (0–7)	1.8 ± 1.7
Any illness in the past 2 weeks	34.5
Number of stimulation activities received by the mother the past 3 days (0–6)	1.6 ± 1.7

We examined biserial correlations among all analysed variables (Supplemental Table 2). As hypothesised, we observed significant correlations between longer birth intervals and child growth and socio-emotional development. However, there were no significant correlations between longer birth intervals and cognitive development, child illness, child dietary diversity, and maternal stimulation.

In the models examining each outcome separately, we found that longer birth intervals were associated with better socio-emotional but not cognitive development (Table 2). Specifically, relative to children with short birth intervals, children with longer birth intervals were more likely to be socio-emotionally on track: RR 1.05 (95% CI 1.00, 1.09). Further, longer birth intervals were associated with significantly higher HAZ: MD 0.23 (0.14, 0.32).

**Table 2:** Associations between longer birth intervals and child growth and development in children 36–59 months of age.

	Cognitive development on track		Socio-emotional development on track		Height-for-age Z-score	
	Unadjusted RR^a^	Adjusted RR^b^	Unadjusted RR^a^	Adjusted RR^b^	Unadjusted MD^a^	Adjusted MD^b^
Preceding birth interval is < 33 months	Ref	Ref	Ref	Ref	Ref	Ref
Preceding birth interval ≥33 months	1.04 (1.00, 1.08)	1.02 (0.98, 1.06)	1.04 (1.00, 1.09)	1.04 (1.00, 1.09)	0.26 (0.17, 0.36)	0.23 (0.14, 0.32)

a Models accounted for clustering and representativeness.

b Models accounted for clustering and representativeness. Estimates controlled for household wealth, location, size, access to improved sanitation, and gender of the household head; maternal age, education, and marital status; child age, sex, whether the child has a twin, number of siblings, child illness and child diet. The models for cognitive and socio-emotional development also controlled for maternal stimulation.

**Fig. 2:**
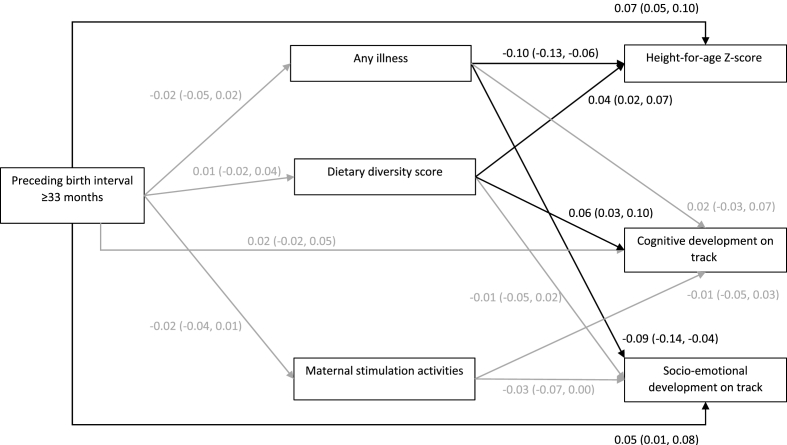
Standardized direct effects and bias-corrected bootstrapped 95% confidence intervals. Grey paths represent estimates not statistically significant at the 5% level. Estimates controlled for household wealth, location, size, access to improved sanitation, and gender of the household head; maternal age, education, and marital status; child age, sex, whether the child has a twin, and number of siblings. The model accounted for clustering and representativeness. Covariances between child illness and child dietary diversity (0.027), child illness and maternal stimulation (0.020), child dietary diversity and maternal stimulation (0.110), HAZ and cognitive development (0.115), HAZ and socio-emotional development (0.031), and cognitive and socio-emotional development (0.048) were estimated but are not depicted in the figure.

Our structural equation model simultaneously estimating all direct, indirect, and total effects showed adequate fit: CFI = 0.988, RMSEA = 0.031, and SRMR = 0.004. After controlling for confounders, longer birth intervals predicted higher HAZ (β = 0.07 (95% CI 0.05, 0.10)) and higher likelihood of on-track socio-emotional development (β = 0.05 (95% CI 0.01, 0.08)), but were not associated with cognitive development (β = 0.02 (95% CI -0.02, 0.05) (Fig. 2). We observed no significant indirect effects on child growth or development via child illness, child dietary diversity, or maternal stimulation (Table 3). Although several indirect effects trended towards significance (e.g., indirect effect between birth interval and socio-emotional development via maternal stimulation), standardized coefficients were close to zero.

**Table 3:** Standardized indirect effects on child growth and development through child illness, child diet, and maternal stimulation.^a^.

	Standardized coefficient (bias-corrected bootstrapped 95% CI)
*Height-for-age Z-score*	
Preceding birth interval ≥33 months → Child illness → Height-for-age Z-score	0.002 (−0.001, 0.005)
Preceding birth interval ≥33 months → Child dietary diversity → Height-for-age Z-score	0.000 (−0.001, 0.002)
*Cognitive development on track*	
Preceding birth interval ≥33 months → Child illness → Cognitive development on track	0.000 (−0.003, 0.000)
Preceding birth interval ≥33 months → Child dietary diversity → Cognitive development on track	0.000 (−0.001, 0.003)
Preceding birth interval ≥33 months → Number of maternal stimulation activities → Cognitive development on track	0.000 (0.000, 0.002)
*Socio-emotional development on track*	
Preceding birth interval ≥33 months → Child illness → Socio-emotional development on track	0.002 (−0.001, 0.005)
Preceding birth interval ≥33 months → Child dietary diversity → Socio-emotional development on track	0.000 (−0.001, 0.000)
Preceding birth interval ≥33 months → Number of maternal stimulation activities → Socio-emotional development on track	0.001 (0.000, 0.002)

a Estimates controlled for household wealth, location, size, access to improved sanitation, and gender of the household head; maternal age, education, and marital status; child age, sex, whether the child has a twin, and number of siblings. The model accounted for clustering and representativeness.

Next, we explored differences in associations across two different groups. First, for maternal education, we compared a constrained model with all parameters equal between children whose mothers had some education and children whose mothers had no education with an unconstrained model where all parameters were freely estimated across the two groups. The latter model was a significantly better fit (p < 0.001). A longer birth interval predicted significantly higher HAZ among children whose mothers had some education (β = 0.114 (95% CI 0.080, 0.151)), but not among children whose mothers had no education (β = 0.036 (95% CI -0.002, 0.072)), though the lower bound of the confidence interval for the latter was just below zero. In addition, better socio-emotional development was predicted by a longer birth interval among children whose mothers had no education: β = 0.074 (95% CI 0.032, 0.119). There were no other significant direct or indirect effects (Supplemental Table 3).

Second, we assessed differences by household wealth. Compared with the constrained model where all parameters were equal between children in poorer households (lowest two wealth quintiles) and children in wealthier households (highest three wealth quintiles), the unconstrained model where all parameters were freely estimated across the two groups was not a significantly better fit (p = 0.305). We observed that a longer birth interval predicted significantly higher HAZ among children in both poorer and wealthier households: β = 0.055 (95% CI 0.018, 0.091) and β = 0.091 (95% CI 0.054, 0125), respectively (Supplemental Table 3). In addition, a longer birth interval predicted better socio-emotional development among children in wealthier households: β = 0.061 (95% CI 0.011, 0.109). No other direct or indirect effects were significant in either group.

## 4 Discussion

We found small-to-moderate associations between longer birth intervals and improved growth and socio-emotional development among children 36–59 months of age in LLMICs. The former findings are consistent with prior studies indicating that short birth intervals increase the risk of child stunting in children under five years of age in LLMICs (Dewey & Cohen, 2007; Fink et al., 2014; Rutstein, 2005; Yaya et al., 2020). Our findings build on a limited literature from high-income countries showing an association between short birth intervals and poorer child cognitive and behavioural development (Crowne et al., 2012; El-Kamary, 2004) by demonstrating an association between birth intervals and socio-emotional development in LLMICs. On the other hand, we found no association between longer birth intervals and cognitive development.

The direct associations we observed between longer birth intervals and child growth and socio-emotional development suggest that there are both biological and behavioural mechanisms underlying this relationship. Longer birth intervals likely influence child growth directly through maternal physiological and biological mechanisms, given the importance of in utero factors for child growth faltering (Danaei et al., 2016), whereas the association with socio-emotional development is likely due to behavioural mechanisms such as increased parental investment and reduced sibling competition for parental resources. The null association with cognitive development may be because birth interval duration is not a risk factor for cognitive development or because of measurement issues.

Through our empirical structural equation model, we found no significant indirect effects on child growth, cognitive or socio-emotional development through child illness, child diet, or maternal stimulation. Statistical significance aside, the magnitude of the coefficients was nearly zero, indicating that the conceptual mechanisms we tested were not empirically supported in this context. Together these findings suggest that longer birth intervals are beneficial for child growth and development and that other biological and behavioural mechanisms appear to drive this relationship. More research is needed to explore additional biological (e.g., birth weight, gestational weight gain) and behavioural (e.g., caregiver mental health, empowerment) mechanisms to better understand the associations between timing of birth intervals and child growth and development. A recent study using DHS data from Ethiopia showed that maternal anaemia and birth size mediated the associations between short birth intervals and child stunting and underweight (Shifti et al., 2022). However, each of these two variables mediated only 4.2–4.5% of the total effect, indicating that other mechanisms are also at work.

In addition, we found that the relationships between longer birth intervals and child growth and socio-emotional development differed for certain groups of children. With respect to child growth, longer birth intervals were more predictive of improved child growth among children whose mothers had some education and those who lived in wealthier households. These results suggest that households with more resources (as proxied by household wealth) and greater caregiver ability to use these resources (as proxied by maternal education) benefit more from longer birth intervals. There may be a threshold effect, such that for longer birth intervals to positively influence child growth, other risk factors for poor child growth need to be addressed and/or eliminated, which may only be possible through higher wealth. The larger association among educated women could also be reflective of some “intentional” decision-making with respect to reproductive health, birth planning, and perinatal health practices that are protective of child growth. With respect to socio-emotional development, we observed significant associations with longer birth intervals among children whose mothers had no education, indicating larger benefits for children exposed to additional maternal risks. At the same time, we observed larger associations between birth intervals and socio-emotional development among children who lived in wealthier households. Similarly, there may be a household wealth threshold to address other risk factors for socio-emotional development, such as inadequate opportunities for learning (Walker et al., 2011). Alternatively, caregivers in wealthier households might be less susceptible to psychosocial risks (e.g., depression and other common mental disorders) stemming from poor socio-economic conditions (Lund et al., 2018) thus enabling improved nurturing care practices. Of note is that these sub-group analyses were exploratory and hypotheses generating. Our results indicate that additional analyses are needed with respect to maternal and household characteristics that might modify the associations between birth intervals, child growth, and development.

A major strength of this study was the use of a large sample that pooled nationally representative data from 13 LLMICs. At the same time, several limitations are worth noting. The DHS data are cross-sectional, which raises potential concerns for reverse causality between the care-related mediators and child outcomes examined in this study. Longitudinal data are needed to ensure the temporal ordering of our conceptual theory of change between birth spacing and early child outcomes. Despite controlling for various sociodemographic characteristics, we could not account for other possible confounders such as maternal prenatal health with respect to the individual child or maternal mental health, as these data are not collected in DHS. Further, nearly all measures were based on maternal self-reports using questionnaires that were relatively brief for the purpose of population-level monitoring. Future studies should further investigate these associations using more psychometrically robust measures, such as validated direct assessments of child development, to reduce measurement bias. Finally, given the limited number of LLMICs in our sample, our findings may not be generalizable to all LLMICs and replication in other LLMICs is warranted.

In conclusion, our study extends global evidence regarding the intergenerational links between birth intervals and early child outcomes, and specifically for child linear growth and socio-emotional development. Our study also expands the literature on older preschoolers 36–59 months of age, an often-neglected group. Our findings highlight the importance of applying a life course perspective that incorporates family planning and reproductive health into the promotion of early child outcomes. Despite exploring a few potential mediating and moderating factors, we found that the relationship between birth intervals and early child outcomes remained largely unexplained by these selected characteristics in our study. Therefore, additional research is needed to investigate alternative biological and behavioural mechanisms as well as other possible effect modifiers. Identifying the underlying processes and elucidating whether there are sub-populations who may be at greater risk can inform the development of more targeted multicomponent reproductive and child health interventions that contain additional components needed to effectively support early child nutrition and development.

## Ethical statement

Ethical clearance for Demographic and Health Surveys (DHS) is granted by the respective countries. DHS data are de-identified secondary data and thus exempt from further ethical review. Access and permission to analyse the data was granted by the DHS program (http://www. dhsprogram.com).

## Funding information

This work was supported by the 10.13039/501100000265Medical Research Council/10.13039/100014013UK Research and Innovation (Grant Ref: MR/T044527/1). For the purpose of open access, the author has applied a Creative Commons Attribution (CC BY) licence to any Author Accepted Manuscript version arising from this submission.

## CRediT author statements

**Conceptualization:** Lilia Bliznashka, Joshua Jeong.

**Data curation:** Lilia Bliznashka.

**Formal analysis:** Lilia Bliznashka.

**Investigation:** Lilia Bliznashka.

**Methodology:** Lilia Bliznashka, Joshua Jeong.

**Supervision:** Lilia Bliznashka.

**Validation:** Joshua Jeong.

**Visualization:** Lilia Bliznashka.

**Writing - original draft:** Lilia Bliznashka.

**Writing - review & editing:** Lilia Bliznashka, Joshua Jeong.

## Declaration of competing interest

The authors declare no conflicts of interest.
