# Supplementary material for: Investigating the direct and indirect associations between birth intervals and child growth and development: A cross-sectional analysis of 13 Demographic and Health Surveys
Source: SSM Popul Health. 2022 Jul 9;19:101168. doi: 10.1016/j.ssmph.2022.101168 (PMC9287629; doi:10.1016/j.ssmph.2022.101168)
Supplement: Multimedia component 1 [file mmc1.docx]

**Supplemental Table 1** Demographic and Health Surveys (DHS) included in the sample

| **Country** | **Phase** | **Year** | **N** |
| --- | --- | --- | --- |
| Benin | Phase VII | 2017-2018 | 1,521 |
| Burundi | Phase VII | 2016-2017 | 958 |
| Cambodia | Phase VII | 2014 | 160 |
| Cameroon | Phase VI | 2011 | 763 |
| Chad | Phase VII | 2014-2015 | 1,551 |
| Congo | Phase VI | 2011-2012 | 548 |
| Haiti | Phase VII | 2016-2017 | 304 |
| Honduras | Phase VI | 2011-2012 | 304 |
| Rwanda | Phase VII | 2014-2015 | 315 |
| Senegal | Phase VII | 2019 | 684 |
| Timor-Leste | Phase-VII | 2016 | 237 |
| Togo | Phase VI | 2013-2014 | 384 |
| Uganda | Phase VII | 2016 | 571 |

**Supplemental Table 2** Unweighted biserial correlations of the variables included in the model

|  | 1 | 2 | 3 | 4 | 5 | 6 | 7 | 8 | 9 | 10 | 11 | 12 | 13 | 14 | 15 | 16 | 17 | 18 |
| --- | --- | --- | --- | --- | --- | --- | --- | --- | --- | --- | --- | --- | --- | --- | --- | --- | --- | --- |
| 1. Preceding birth interval >=33 mo |  |  |  |  |  |  |  |  |  |  |  |  |  |  |  |  |  |  |
| 2. Height-for-age Z-score | 0.10** |  |  |  |  |  |  |  |  |  |  |  |  |  |  |  |  |  |
| 3. Cognitive development on track | 0.02 | 0.13** |  |  |  |  |  |  |  |  |  |  |  |  |  |  |  |  |
| 4. Socio-emotional development on track | 0.03* | 0.02* | 0.01 |  |  |  |  |  |  |  |  |  |  |  |  |  |  |  |
| 5. Child had any illness | 0.00 | -0.06** | 0.02 | -0.06** |  |  |  |  |  |  |  |  |  |  |  |  |  |  |
| 6. Child dietary diversity score | 0.02 | 0.06** | 0.08** | -0.02 | 0.03** |  |  |  |  |  |  |  |  |  |  |  |  |  |
| 7. Number of maternal stimulation activities | -0.02 | -0.01 | -0.01 | -0.02 | 0.02* | 0.12** |  |  |  |  |  |  |  |  |  |  |  |  |
| 8. Child age | -0.04** | -0.01 | 0.08** | -0.02* | -0.02 | 0.05** | -0.02* |  |  |  |  |  |  |  |  |  |  |  |
| 9. Child sex | -0.01 | -0.03* | -0.03** | -0.07** | 0.00 | 0.02 | -0.02* | 0.00 |  |  |  |  |  |  |  |  |  |  |
| 10. Child had a twin | 0.02* | -0.04** | -0.02* | -0.03* | -0.01 | -0.01 | 0.00 | 0.00 | -0.02 |  |  |  |  |  |  |  |  |  |
| 11. Number of living siblings | -0.03* | -0.01 | 0.02 | -0.02* | 0.00 | -0.01 | -0.01 | -0.02* | -0.01 | 0.02 |  |  |  |  |  |  |  |  |
| 12. Maternal age | 0.12** | 0.05** | 0.03** | 0.01 | 0.00 | 0.01 | -0.05** | 0.08** | -0.01 | 0.02 | 0.03** |  |  |  |  |  |  |  |
| 13. Maternal education | 0.04** | 0.13** | 0.11** | -0.02* | 0.07** | 0.13** | 0.18** | -0.03* | 0.01 | 0.01 | 0.00 | -0.05** |  |  |  |  |  |  |
| 14. Mother was married/co-habitating | -0.03** | 0.02 | -0.03** | -0.01 | -0.02* | -0.02* | -0.01 | -0.01 | 0.00 | 0.00 | 0.03* | 0.00 | -0.07** |  |  |  |  |  |
| 15. Household size | -0.03* | 0.06** | 0.01 | 0.01 | -0.06** | -0.03** | -0.12** | 0.00 | -0.01 | 0.01 | 0.03** | 0.14** | -0.12** | 0.02 |  |  |  |  |
| 16. Female-headed household | 0.05** | 0.04** | 0.02* | -0.01 | 0.01 | 0.01 | -0.01 | 0.00 | 0.01 | -0.01 | -0.03** | 0.03** | 0.04** | -0.31** | -0.1** |  |  |  |
| 17. Household lived in rural area | -0.03** | -0.14** | -0.07** | -0.02* | 0.06** | -0.06** | -0.07** | 0.02 | 0.00 | -0.02 | 0.02 | 0.03* | -0.19** | 0.01 | -0.01 | -0.02 |  |  |
| 18. Household wealth | 0.01 | 0.12** | 0.04** | 0.00 | -0.04** | 0.07** | 0.14** | 0.01 | 0.01 | 0.01 | -0.02* | -0.01 | 0.23** | 0.06** | 0.02 | -0.01 | -0.45** |  |
| 19. Improved sanitation | 0.01 | 0.07** | 0.08** | 0.02 | 0.02 | 0.09** | 0.06** | 0.01 | -0.01 | -0.01 | -0.03** | 0.05** | 0.15** | 0.02 | 0.08** | 0.02 | -0.13** | 0.28** |

* *p*<0.05; ** *p*<0.01

**Supplemental Table 3** Standardized direct effects on child growth and development and indirect effects through child illness, child diet, and maternal stimulation by maternal education and household wealth groupings^1^

|  | **Mother had some education** | **Mother had no education** | **Household was in the lowest two wealth quintiles** | **Household was in the highest three wealth quintiles** |
| --- | --- | --- | --- | --- |
| *Height-for-age Z-score* |  |  |  |  |
| *Direct effect* |  |  |  |  |
| Preceding birth interval ≥33 months → Height-for-age Z-score | 0.114  (0.080, 0.151) | 0.036  (-0.002, 0.072) | 0.055  (0.018, 0.091) | 0.091  (0.054, 0.125) |
| *Indirect effects* |  |  |  |  |
| Preceding birth interval ≥33 months → Child illness → Height-for-age Z-score | 0.000  (-0.004, 0.004) | 0.004  (-0.001, 0.011) | 0.004  (-0.002, 0.011) | 0.000  (-0.004, 0.004) |
| Preceding birth interval ≥33 months → Child dietary diversity → Height-for-age Z-score | 0.000  (-0.001, 0.001) | 0.002  (-0.001, 0.005) | 0.001  (-0.001, 0.004) | 0.000  (-0.001, 0.002) |
| *Cognitive development on track* |  |  |  |  |
| *Direct effect* |  |  |  |  |
| Preceding birth interval ≥33 months → Cognitive development on track | 0.025  (-0.029, 0.080) | 0.008  (-0.038, 0.054) | 0.004  (-0.042, 0.046) | 0.027  (-0.029, 0.088) |
| *Indirect effect* |  |  |  |  |
| Preceding birth interval ≥33 months → Child illness → Cognitive development on track | 0.000  (-0.003, 0.004) | 0.000  (-0.002, 0.004) | 0.000  (-0.002, 0.003) | 0.000  (-0.004, 0.002) |
| Preceding birth interval ≥33 months → Child dietary diversity → Cognitive development on track | 0.000  (-0.003, 0.001) | 0.001  (-0.001, 0.006) | 0.001  (-0.001, 0.003) | 0.000  (-0.004, 0.004) |
| Preceding birth interval ≥33 months → Number of maternal stimulation activities → Cognitive development on track | 0.000  (-0.004, 0.001) | 0.000  (-0.001, 0.003) | 0.000  (-0.001, 0.003) | 0.000  (-0.003, 0.001) |
| *Socio-emotional development on track* |  |  |  |  |
| *Direct effect* |  |  |  |  |
| Preceding birth interval ≥33 months → Socio-emotional development on track | 0.015  (-0.032, 0.063) | 0.074  (0.032, 0.119) | 0.032  (-0.009, 0.074) | 0.061  (0.011, 0.109) |
| *Indirect effect* |  |  |  |  |
| Preceding birth interval ≥33 months → Child illness → Socio-emotional development on track | 0.000  (-0.005, 0.005) | 0.002  (0.000, 0.009) | 0.003  (-0.001, 0.009) | 0.000  (-0.004, 0.004) |
| Preceding birth interval ≥33 months → Child dietary diversity → Socio-emotional development on track | 0.000  (-0.001, 0.001) | -0.001  (-0.003, 0.000) | 0.000  (-0.003, 0.000) | 0.000  (-0.001, 0.001) |
| Preceding birth interval ≥33 months → Number of maternal stimulation activities → Socio-emotional development on track | 0.001  (-0.001, 0.005) | 0.000  (-0.002, 0.001) | 0.000  (-0.001, 0.003) | 0.001  (0.000, 0.004) |

^1^ Estimates are standardized coefficients with bias-corrected bootstrapped 95% CI. Estimates controlled for household wealth, rurality, size, access to improved sanitation, and gender of the household head; maternal age, education, and marital status; child age, sex, whether the child has a twin, and number of siblings. The model accounted for clustering and representativeness.
